# The Differences Between Same-Day and Staged (Circumferential) Fusion Surgery in Adult Spinal Deformity: Protocol for a Systematic Review

**DOI:** 10.2196/42331

**Published:** 2022-11-28

**Authors:** Mert Marcel Dagli, Shivek Narang, Kashish Malhotra, Gabrielle Santangelo, Connor Wathen, Yohannes Ghenbot, Dominick Macaluso, Ahmed Albayar, Ali Kemal Ozturk, William C Welch

**Affiliations:** 1 Department of Neurosurgery Perelman School of Medicine University of Pennsylvania Philadelphia, PA United States; 2 Department of Surgery Dayanand Medical College and Hospital Ludhiana India

**Keywords:** spinal surgery, scoliosis, kyphosis, protocol, circumferential, adult spinal deformity, differences, fusion, fusion surgery, spinal curvature, spine deformity, spinal deformity, surgery, surgical, perioperative, review methodology, systematic review, search strategy, protocol

## Abstract

**Background:**

Adult spinal deformity (ASD) is a deformity in the curvature of the adult spine. ASD includes a range of pathology that leads to decreased quality of life for patients as well as debilitating morbidities. Treatment can range from nonoperative management to long-segment surgical corrections and depends greatly on the deformity and patient profiles. If surgical treatment is indicated, circumferential (a combined anterior and posterior approach) fusion is one of the tools in the spine surgeon’s armamentarium. Depending on the complexity, the procedure is either completed on the same day or staged. Determining whether to perform a circumferential surgery in a staged fashion is based largely on the surgeon’s preference and perception of the individual case complexity; at present, there is no high-quality evidence that can be used to support that decision.

**Objective:**

This paper presents the protocol for a systematic review that aims to investigate the differences between same-day versus staged circumferential fusion surgery in ASD both in patient selection and in outcomes.

**Methods:**

Searches will be performed on MEDLINE, Embase, the Cochrane Central Register of Controlled Trials, Web of Science, and Scopus. Gray literature and the reference lists of articles included in the full-text screening will also be screened for inclusion. Results will be exported to Covidence. Data will be collected on demographics, type of procedures performed, surgery levels, blood loss, total operation time, length of stay, disposition, readmissions (30 days and 90 days), and perioperative complications. Patient-reported outcomes will also be assessed. Data quality assessment of randomized controlled trials will be performed using the Cochrane Collaboration’s tool for assessing risk of bias in randomized trials, and nonrandomized studies will be assessed with the ROBINS-I (Risk of Bias in Non-randomized Studies of Interventions) tool. All screening, quality assessment, and data extraction will be done by 2 independent reviewers. A descriptive synthesis will be performed, and data will be evaluated for further analysis.

**Results:**

This study is currently in the screening phase. There are no results yet. The search strategy has been developed and documented. Information has been exported to Covidence. Upon conclusion of the critical appraisal stage, screening and extraction, as well as a synthesis of the results, will be performed.

**Conclusions:**

The intended review will summarize the differences in perioperative outcomes and complications between same-day and staged (circumferential) fusion surgery in adult spinal deformity. It will also describe the patients selected for such procedures based on their demographics and pathology. Identified gaps in knowledge will provide insight into current limitations and guide further studies on this topic.

**Trial Registration:**

PROSPERO CRD42022339764; https://www.crd.york.ac.uk/prospero/display_record.php?RecordID=339764

**International Registered Report Identifier (IRRID):**

PRR1-10.2196/42331

## Introduction

### Background

Adult spinal deformities (ASDs) are defined as abnormalities in the spinal curvature or alignment in the adult population that deviate from normal limits [1]. ASD can include any combination of spinal deformities, such as kyphosis, lordosis, and scoliosis. ASD is becoming more prevalent with the increasing age of the population [1-4]. Once conservative management has failed, surgical correction is considered. Common indications for surgery are pain with substantial abnormality in spinal curvature, significant deformities that are esthetically unacceptable to the patient, documented curve progression with imbalance in one or more planes, and significant loss of pulmonary function attributed to the deformity [5-9].

Depending on the complexity and patient-specific surgical risk profile, ASD surgeries, such as circumferential procedures, can be done on the same day or staged and completed on a different date [10-17]. Differences in outcome between same-day and staged surgery have been a topic of interest for surgeons.

### Rationale and Objective

To our knowledge, no systematic review of published literature on this topic has been performed. Our study aims to shed light on the current literature, highlight limitations, identify gaps in knowledge, and guide future studies on the management of ASD with either same-day or staged circumferential fusion.

## Methods

### Protocol and Registration

The protocol was developed based on the PRISMA-*P* 2015 (Preferred Reporting Items for Systematic Review and Meta-Analysis Protocols) methodology (see checklist in Multimedia Appendix 1) [18,19]. The protocol is registered in PROSPERO (International Prospective Register of Systematic Reviews; CRD42022339764).

### Eligibility Criteria

The PICO (population, intervention, comparison, and outcome) framework was used to formulate the eligibility criteria:

- Population: patients with adult spinal deformity;
- Intervention: staged (circumferential) fusion surgery;
- Comparison: same-day (circumferential) fusion surgery;
- Outcome: differences in perioperative outcomes, complications, length of stay, disposition, readmissions, and patient-reported outcomes.

### Inclusion and Exclusion Criteria

We will include all clinical studies of patients with ASD who underwent staged (circumferential) fusion surgery. Studies that include nonhuman subjects or a nonadult population, compare different types of surgery that do not differ in timing (same day vs staged), case reports, case series, studies presenting a technical report of the procedure performed without reporting any original data, and conference abstracts will be excluded. Additionally, only literature in English will be considered.

### Search Strategy

A comprehensive systematic search strategy has been developed in conjunction with an external librarian. MEDLINE, Embase, the Cochrane Central Register of Controlled Trials, Web of Science, and Scopus will be searched. We will also search Google Scholar for gray literature and screen the references of articles included in the full-text screening for inclusion in our systematic review. A sample search strategy specific to MEDLINE has been generated and is presented in Textbox 1, including database-specific search information, such as controlled vocabulary and keywords. All results will be exported and deduplicated on Covidence [20].

**Complete search strategy for MEDLINE.**

- Search #1: (“spinal curvatures”[MeSH Terms] OR “spinal curvatures”[MeSH Terms] OR “adult spinal deformity”[tiab] OR “adult degenerative deformity”[tiab] OR “asd”[tiab] OR “spinal deformity”[tiab])
- Search #2: (“staging”[tiab] OR “staged”[tiab] OR “same day”[tiab] OR “stag*”[tiab])
- Search #3: (“circumferential”[tiab] OR “anterior posterior”[tiab] OR (“anterior”[tiab] AND “posterior”[tiab]) OR “posterior”[tiab] OR “anterior”[tiab])
- Search #4: (“fusion”[tiab] OR “spinal fusion”[tiab] OR “spinal surgery”[tiab] OR “spinal fusion surgery”[tiab])
- (#1 AND #2) OR (#2 AND #3 AND #4)

### Data Selection and Extraction

Two independent reviewers will participate in a title and abstract screen on Covidence. A third reviewer will resolve any disagreements. After completion of the title and abstract screen, the results will be exported to EndNote 20 (Clarivate), and institutional access will allow for automatic integration of the full-text PDFs [21]. Thereafter, the references will be reimported to Covidence. Full-text review will commence, and data extraction will subsequently be performed.

Key data for extraction will include, but will not be limited to, study information (first author and date of publication), study design, number of participants included in the study, demographics, type of procedures being performed, surgery levels, blood loss, total operation time, length of stay, disposition, readmissions (30 days and 90 days), patient-reported outcomes (eg, the Neck Disability Index, the Oswestry Disability Index, and EQ-5D), intraoperative complications (eg, intensive care unit admissions and stays), and postoperative complications (eg, medical, surgical) [22-24].

### Data Quality

The methodological quality and risk of bias of eligible studies will be critically appraised by 2 independent reviewers. A data quality assessment of randomized controlled trials will be performed using the Cochrane Collaboration’s tool for assessing risk of bias in randomized trials [25]. Nonrandomized studies will be assessed with the ROBINS-I (Risk of Bias in Non-randomized Studies of Interventions) tool [26].

### Data Synthesis

Due to the nature of this review and expected paucity of data, a descriptive synthesis will be performed. Therefore, data will be presented descriptively in tables. Additionally, graphical formats will be used as appropriate. This is subject to change depending on the extracted data. An internal statistician will evaluate a best-practice approach.

## Results

This study is in the critical appraisal stage. No results have been obtained yet. At the time of writing, the developed search strategy had been used. Information from databases has been extracted to Covidence and records have been deduplicated. The screening stage has not concluded yet.

## Discussion

To our knowledge, this will be the first systematic review on the differences between same-day and staged circumferential fusion surgery in ASD focusing on the current evidence and its limitations. The decision to stage a surgery for a complex deformity case comes with certain tradeoffs for the surgeon and patient. Some surgeons prefer to minimize complexity by staging and, in theory, minimize the morbidity associated with long operative and anesthesia times. Others elect to combine approaches on the same day to theoretically limit anesthesia events and blood loss, reduce total operative time, and reduce the overall length of stay and hospital costs.

The available literature on staging ASD procedures is limited by small sample sizes and inclusion of diverse pathologies (degenerative, infectious, neoplastic, or traumatic), making interpretation difficult. Nearly 30 years ago, Shufflebarger et al [27] reported a retrospective review of staged (n=35) versus same-day (n=40) surgery for ASD that showed significantly less total blood loss, lower postoperative complication rates, and a more favorable deformity correction. Another small retrospective study of 11 patients per group showed that same-day surgeries were associated with less blood loss, decreased postoperative morbidity, and shorter lengths of stay [28]. With regard to extended hospitalization, Stephens et al [29] demonstrated that it is independently associated with increased costs after ASD surgeries. A national population-based discharge database was used to analyze outcomes in 11,265 circumferential spine surgeries with a subgroup analysis of same-day versus staged procedures. The staged group was associated with increased perioperative complications, including postoperative venous thrombosis and acute respiratory distress syndrome [30]. The authors then performed a propensity-matched analysis of a retrospective cohort comparing same-day versus staged spine surgery in ASD with similar complication rates between groups. However, the staged group also required more revision surgery at the 2-year follow-up than the same-day group [16].

A limitation of this study is the relative paucity of high-quality evidence in this domain given the retrospective nature of many studies investigating this issue. Additionally, there are external factors that may influence the decision to perform same-day or staged surgery, such as surgical training, operating room availability, organizational practice patterns, and patient preference, which cannot be directly studied in this review.

Our systematic review will provide surgeons with a rigorous analysis of the available data on same-day versus staged procedures for circumferential fusion. The decision to stage a procedure has thus far been largely driven by the individual surgeon’s practice patterns or because of the complexity of a patient’s deformity or medical comorbidities. With the aging population and the increase in ASD, evidence-based practice will promote the best outcomes for our patients and avoid unnecessary and costly complications. Understanding the literature available at this point and its limitations will help to guide future prospective trials to deepen our understanding of this complex problem.

## Appendix

Multimedia Appendix 1 The PRISMA-P (Preferred Reporting Items for Systematic Review and Meta-Analysis Protocols) checklist.

## Abbreviations

ASD: adult spinal deformity
PICO: population, intervention, comparison, and outcome
PRISMA-P: Preferred Reporting Items for Systematic Review and Meta-Analysis Protocols
PROSPERO: International Prospective Register of Systematic Reviews
ROBIN-I: Risk of Bias in Non-randomized Studies of Interventions
